# A machine learning approach to quantify gender bias in collaboration practices of mathematicians

**DOI:** 10.3389/fdata.2022.989469

**Published:** 2023-01-18

**Authors:** Christian Steinfeldt, Helena Mihaljević

**Affiliations:** Department 4 - Computer Science, Communication and Economics, Hochschule für Technik und Wirtschaft Berlin, University of Applied Sciences, Berlin, Germany

**Keywords:** collaboration networks, machine learning, gender in mathematics, regression-based analysis, authorship, scientific publishing, single-authored publications, coauthorship

## Abstract

Collaboration practices have been shown to be crucial determinants of scientific careers. We examine the effect of gender on coauthorship-based collaboration in mathematics, a discipline in which women continue to be underrepresented, especially in higher academic positions. We focus on two key aspects of scientific collaboration—the number of different coauthors and the number of single authorships. A higher number of coauthors has a positive effect on, e.g., the number of citations and productivity, while single authorships, for example, serve as evidence of scientific maturity and help to send a clear signal of one's proficiency to the community. Using machine learning-based methods, we show that collaboration networks of female mathematicians are slightly larger than those of their male colleagues when potential confounders such as seniority or total number of publications are controlled, while they author significantly fewer papers on their own. This confirms previous descriptive explorations and provides more precise models for the role of gender in collaboration in mathematics.

## 1. Introduction

Nowadays, research is built as group effort, in which individuals collaborate through joint discussions of ideas and methods, oral and written presentations, and the integration of obtained feedback into further work (Ductor et al., 2018). It is thus not so surprising that the notion of mathematics as a discipline pursued by individual geniuses is considered outdated. But even historically, mathematics offers a range of examples of fruitful collaborations. The presumably most prominent such example are Hardy and Littlewood, who jointly wrote about 100 papers of great importance for pure mathematics in England in the first half of the twentieth century (Wilson, 2002). Paul Erdős, one of the most prolific mathematicians in history, helped to transform the discipline into a social activity by collaborating with more than 500 coauthors. The public forum-based collaboration on the Hales-Jewett theorem, driven by Tim Gowers' *Polymath* project, has proven that even “massively collaborative mathematics” is possible (Gowers, 2009).

Data on coauthorship confirms the trend toward collaboration through joint authorship of scientific publications. According to Mathematical Reviews, the percentage of papers with multiple authors increased from 9% in the 1940s to 46% in the 1990s (Grossman, 2002). These figures agree well with those of zbMATH, suggesting that currently about three quarters of all publications in mathematics are written collaboratively (Mihaljević and Santamaría, 2020). Writing papers with other scholars increases one's visibility within the research community and can thus advance the academic career. For example, research has shown for various disciplines that the network size, i.e., the number of one's distinct coauthors, is positively correlated with a larger number of citations (Wuchty et al., 2007; Sarigöl et al., 2014; Servia-Rodríguez et al., 2015) and higher productivity (Ductor, 2015; Servia-Rodríguez et al., 2015). At the same time, publishing in groups reduces various risks, such as openly hostile criticism or the responsibility for errors (Kwiek and Roszka, 2022).

Nevertheless, a successful academic career, in mathematics and other disciplines, is typically built on both collaborative and individual work. In past forecasts, single authorships have generally been seen by numerous researchers as somewhere between decline and extinction (Price, 1963; Allen et al., 2014; Barlow et al., 2018; Kuld and O'Hagan, 2018; Ryu, 2020). Despite the increase in collaborations and the accompanying decrease in single-authored papers, this prediction has not come true. In some disciplines such as humanities and literature, but also mathematics, papers written by one individual still account for a significant share, if not the majority of all published research. Single authorships fulfill a certain function that cannot be replaced by collaboration. They serve as proof of one's ability and credibility as a scientist, showing that one is not “dependent on senior people for ideas, guidance, techniques, […] Hence, one is ready for a faculty position” (McKenzie, 2012). This makes solo publications especially valuable at an early career stage (Kuld and O'Hagan, 2018), sending a clear signal to the academic job market. Moreover, in contrast to collaboration, writing a paper alone does not require making compromises, it comes without unclear responsibilities and communication issues commonly referred to as “coordination costs” (Olechnicka et al., 2020; Kwiek and Roszka, 2022), and it is not affected by unclear credit attribution. The latter has shown to be associated with strong gender bias for research in economics, putting female economists collaborating with men at a disadvantage (Sarsons et al., 2021).

Given the importance of collaboration practices for the pursuit of one's research and ultimately the trajectory of academic careers, the question of the role of gender in relation to network parameters arises. Although the number of women starting to publish in STEM fields is steadily growing, they tend to have shorter careers (see, e.g., Boekhout et al., 2021), and their proportion progressively decreases when it comes to high-level academic positions, in particular tenured posts (cf. e.g., Golbeck et al., 2018; European Commission, Directorate-General for Research and Innovation, 2019). Thus, we ask whether there are gender-based differences in the size of coauthor networks and the proportion of single authorships. In previous research on mathematics (Mihaljević-Brandt et al., 2016), we showed that men write 38% of their scientific records as single authors, in contrast to 29% for women. This trend remained stable even after grouping authors into seven segments based on their total number of publications. At the same time, the network sizes of female and male mathematicians turned out to be similar. However, the analyses were descriptive, the segments reflecting the number of publications were relatively rough, and further potential confounders such as seniority were not considered—thus not allowing to conclude that there is something intrinsic to men's and women's collaboration patterns in mathematics.

In this paper, we investigate the two target variables *network size* and *number of single authorships* using appropriate comparative models, which allow us to further isolate and quantify the effect of gender. We follow two machine learning based approaches to quantify model bias and compare their results in order to obtain a robust assessment of the role of the gender variable. The models are trained using data from zbMATH Open, one of the most comprehensive indexing and reviewing services for mathematics (FIZ Karlsruhe, 2022). We show that women in mathematics have similar, even slightly higher, numbers of distinct coauthors as men when we control for the number of publications, time-based variables such as publication year and seniority, the subfield, perceived journal quality and the continent of the author's affiliation. At the same time, we show that the number of single authorships is lower, by around 4.5% with respect to the overall number of publications, even after controlling for the aforementioned potential confounders. This difference, while notable, is still less than suspected based on existing research.

## 2. Related work

A number of studies have examined network aspects of coauthorship in relation to gender, with varying results depending on the discipline studied and the underlying dataset. In terms of the size of coauthor networks, depending on the study and the modeling approach, sometimes men and sometimes women are seen in front, although usually the observed difference is rather small.

Jadidi et al. (2018) deduce from DBLP data that women and men computer scientists exhibit some structural differences when building coauthorship networks. In particular, men develop significantly larger networks, even when controlling for time variables such as the year or seniority, though with small effect size. In a recent descriptive analysis of major conferences in computer systems in 2017 (Yamamoto and Frachtenberg, 2022), men were shown to have more coauthors per paper and overall than women, but the difference was rather small. An analysis of four Italian conferences in the fields of information systems and computer science revealed that “men are more key than women” in only one of the four considered communities. Although men were shown to have more connections than women in three communities in terms of degree and degree centrality, women had “similar values of betweenness, eigencentrality and closeness and, hence, similar probability to diffuse topics. This means that women tend to connect more with key members with respect to what men do” (De Nicola and D'Agostino, 2021). A study analyzing the complete publication records of almost 4,000 faculty members in six STEM disciplines at selected research universities in the U.S. revealed that “female faculty have significantly fewer distinct coauthors over their careers than males, but that this difference can be fully accounted for by females' lower publication rate and shorter career lengths” (Zeng et al., 2016). A slightly older survey-based study by Bozeman and Gaughan (2011) analyzed responses from 1,714 tenured and tenure track faculty members at Carnegie research extensive universities, working in STEM disciplines. Their models, taking into account factors such as tenure, discipline, family status, and doctoral cohort, indicate that “women actually have somewhat more collaborators on average than do men” (Bozeman and Gaughan, 2011). The authors further show that interaction with industrial partners and research centers is positively correlated with the number of collaborators. Pina et al. (2019) studied a sample of junior and senior life science grantees from the European Research Council (ERC) from the years 2007 to 2009 regarding publication and citation outputs and collaboration networks with respect to gender, seniority, and country of work. The authors were “particularly interested in the change of publication performance in relation to the grant award” and thus studied the 5-year period before and after the award was received. Almost no gender differences were observed related to scientific networking, the only exception being a greater network size after grant award for male junior grantees.

While the cited studies discover rather minor differences with respect to the number of distinct coauthors, Ductor et al. draw a more contrasting picture for economy. Based on an extensive analysis of 1,627 journals in economics between 1970 and 2011, they “identify large and persistent gender differences” in coauthorship networks. Female economists have a lower number of distinct coauthors, and the difference increased over time. Moreover, they show that women “co-author more with more experienced and senior economists” (Ductor et al., 2018). The authors deduce from their overall results that the differences in building networks through coauthorship stem from differences in risk-taking that in turn could be explained by disparities in preferences or the environment, e.g., differences in rewards for the same type of action. This fits well with the results by Sarsons et al. (2021), who show for economists that “an additional coauthored paper is correlated with an 7.4% increase in tenure probability for men but only a 4.7% increase for women.” The latter gap turns out to be less pronounced in collaborations among women, indicating that attribution of credit for group work is related to the gender of coauthors, so that in mixed-gender collaborations, mainly men receive the credit for the joint work.

Single authorships have unfortunately been less addressed in previous research, despite being a relevant career factor whose effect on career advancement might, in contrast to coauthorship, be less dependent of authors' gender. As shown by Sarsons et al. (2021), female and male economists who write most of their papers alone have similar tenure rates, conditional on the quality of their contributions. Existing studies on sole authorship seem to agree that women write a smaller proportion of their publications alone (West et al., 2013; Ductor et al., 2018; Sarsons et al., 2021; Yamamoto and Frachtenberg, 2022). In one of the few works addressing the question of gender and solo research in more detail, however, Kwiek and Roszka (2022) find only marginal gender differences among researchers at Polish universities. They formally introduce the gender solo research gap and extensively study its underlying hypothesis that “female scientists are less involved in publishing alone than male scientists.” They find statistically significant gender differences only among young academics, and with rather small effect. Overall, the strongest predictor of individual solo publishing rate is the overarching scientific discipline and their collaboration practices in terms of average team-size (STEM fields and international cooperation negatively affect the rate, while publishing in male-dominated disciplines positively affects it). A similar discipline-specific difference is also observed by Farber (2005) within Israeli universities, where single authorships are more likely in theoretical research. Based on publications in accounting and finance, Vafeas (2010) shows that the likelihood of solo authorships is higher for conceptual or analytical projects rather than empirical ones, but also, e.g., “when the author is affiliated with a highly ranked university.”

Specifically for mathematics, the questions of coauthorship network sizes and the proportion of single authorships is investigated in Mihaljević-Brandt et al. (2016). Based on data from zbMATH covering a period of more than 40 years, differences were found in the proportion of single authorships between men and women. Even after grouping all authors into six segments based on the total number of published works, a stable overall difference of almost 10% is observed. At the same time, there is almost no difference in terms of network sizes, with slightly higher mean and median values for women in some of the segments. However, the work does not take into account further variables such as seniority, publication year, or mathematical subfield—all being potential confounders for the respective target variables.

## 3. Data and methods

### 3.1. Data source, variables, and format

Our analysis is based on data from the freely accessible repository *zbMATH Open* (former Zentralblatt MATH), “the world's most comprehensive and longest-running abstracting and reviewing service in pure and applied mathematics” (FIZ Karlsruhe, 2022). As of April 2022, the service comprises about 4.2 million entries written by more that 1.1 Million different authors.

zbMATH Open indexes publications from all areas of pure and applied mathematics, their applications, history, and philosophy of mathematics and mathematics university education. The broad coverage of zbMATH results in a partial inclusion of scientists whose main expertise belongs to other disciplines such as physics or computer science. We thus restrict to publications by so-called *core mathematicians*, roughly defined as individuals who have published at least one article in a journal with clear mathematics focus; for a definition of the respective heuristics (see Mihaljević and Santamaría, 2020). From this data, we extract the following variables that we consider relevant to the task at hand and build our final dataset.

We consider two authorship-related target variables—the *network size*, where network refers to the ego-network of an author within the coauthorship network in which edges are drawn between two author nodes, if they coauthored a joint publication (and thus equals the number of distinct coauthors of a given mathematician), and the *number of single authorships*. To measure the relevance of gender to the target variables, we build predictive models for the annual cumulative achievements of every author. More specifically, each record in our dataset represents the network size as well as the number of solo publications of an author at the end of a given year. Accordingly, the author ID^1^ and the year provide a unique identifier.

**Figure 1:**
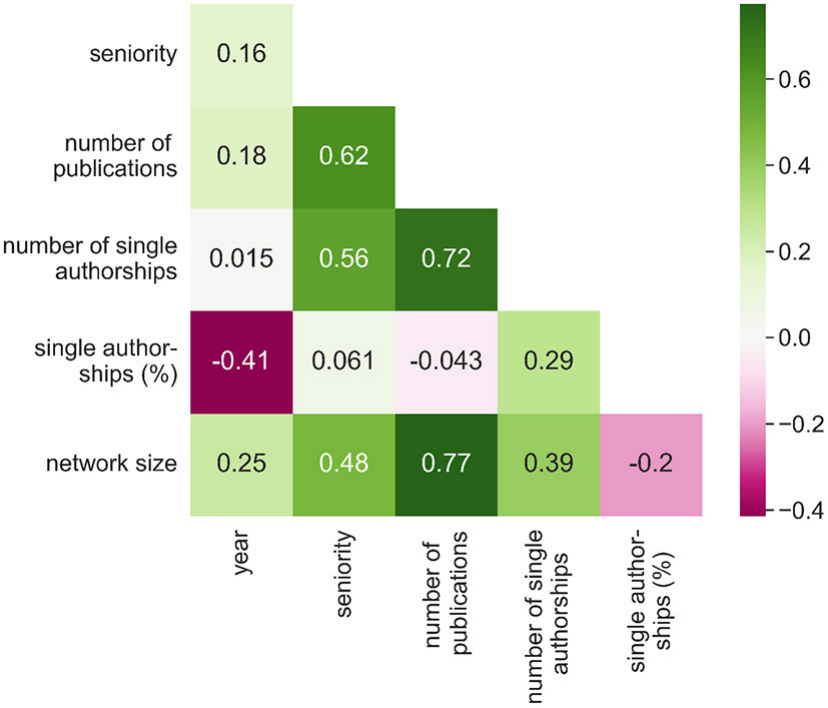
Pairwise Pearson correlation coefficients between numerical input variables and target variables. For better understanding, we include the percentage of single authorships among all authorships of an author. All relationships are statistically significant with *p*-value 0.01.

We build the prediction models using multiple features that we consider relevant for the task at hand. In addition to the *number of publications*, we compute the *seniority* as the number of years since the author's first publication. Figure 1 shows that these two variables have the highest correlation coefficient with both target variables (Since reprints, obituaries, and similar publications appear after an author's death but are also associated with the respective author profile in zbMATH, we excluded all publications after a gap of 9 years. Tests on sample data show that the procedure is robust and yields plausible results).

To give an example, suppose an author *A* starts publishing in 2015 by jointly writing two papers with the same coauthor *B*. In the subsequent years 2016 and 2017, *A* has no further publication activity. In 2018, *A* returns with a single-authored paper, as well as a collaboration with a group of 4 new coauthors, concluding this author's publication career. In our dataset, author *A* would be modeled with the records displayed in Table 1.

**Table 1:** Representation of selected variables using all records of a fictitious author *A* as an example.

**ID**	**Year**	**Number of publications**	**Seniority**	**Network size**	**Number of single authorships**
A	2015	2	0	1	0
A	2016	2	1	1	0
A	2017	2	2	1	0
A	2018	4	3	5	1

**Figure 2:**
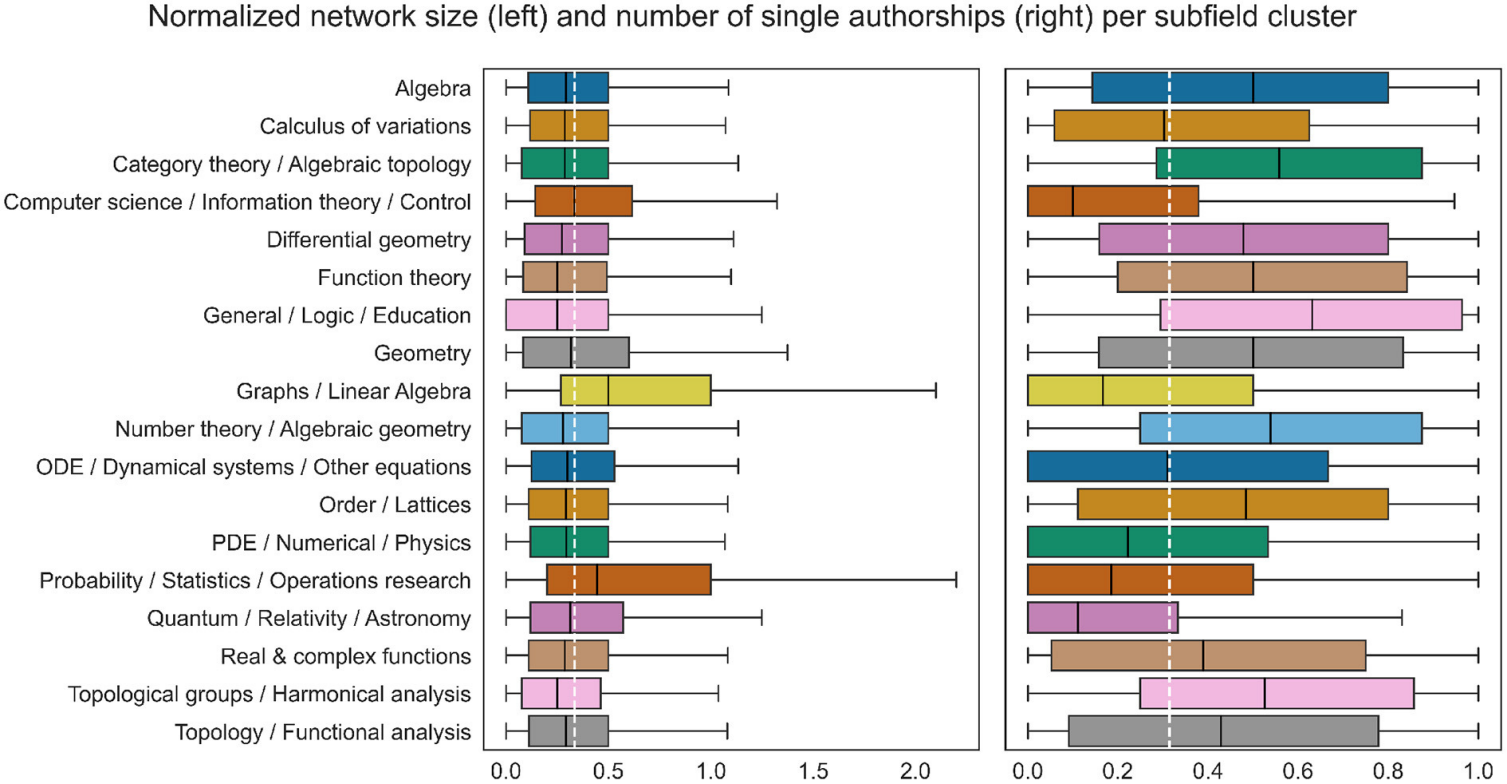
Differences in the value distributions of the target variables between subfield clusters. The target variables were normalized by dividing each by the total number of publications, as the latter is strongly correlated with the target variables and at the same time subfield specific. The white dashed line marks the normalized mean of the entire data. The overall difference is statistically significant with *p*-value 0.01, measured with one-way ANOVA.

Publication practices are subfield specific. zbMATH provides codes reflecting the topics of the publications based on MSC2010, a hierarchical tree-based scheme with 63 codes on the highest level^2^. To decrease the granularity, we previously built a data-driven clustering of MSC codes, yielding 18 *subfield clusters* altogether. To every record in our dataset we assign the most frequent subfield cluster among the considered publications. Figure 2 presents Box-Whisker plots of both target variables across the subfield clusters, showing a significant variation among the clusters in particular with respect to the proportion of single-authored publications.

**Figure 3:**
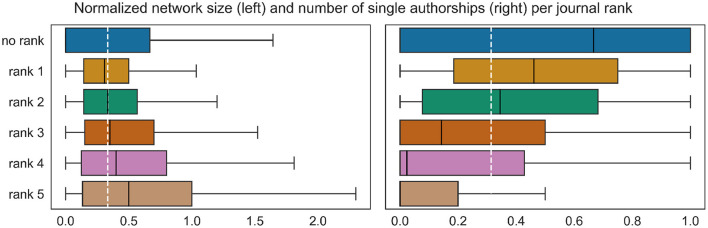
Differences in the value distributions of the target variables between journal ranks. The target variables were normalized as in Figure 2. The white dashed line marks the normalized mean of the entire data. The overall difference is statistically significant with *p*-value 0.01, measured with one-way ANOVA.

In addition, we make use of zbMATH's scheme consisting of five *journal ranks* to prioritize publication venues indexed by the service. We resort to their internal scheme since it is updated on a regular basis by zbMATH's editorial staff and a group of experts from various subfields, while there is no public categorization recognized by the mathematical community (cf. Mihaljević and Santamaría, 2020). Note that some older journals that are not published anymore do not have a rank. As for subfield clusters, to every record we assign the most frequent journal rank among the considered publications. Figure 3 shows a clear correlation between journal ranks and both target variables: authors publishing predominantly in journals with higher rank have larger coauthorship networks, while rank 1 and in particular the old journals without a rank are rather associated with smaller network sizes. The tendency is in the opposite direction for the second target variable.

Network building is further related to the country of work (cf. e.g., Pina et al., 2019; Mihaljević and Santamaría, 2020, p. 106). Affiliations containing geographic information are not complete in zbMATH Open, with gaps being most pronounced for older publications. To reduce the number of missing values and to simplify the data model, we (1) reduce the granularity from country to continent and (2) keep the first found continent per author for all years, as rather little migration from one continent to another can be observed in the data. In the final dataset, a location is missing for ~25% of all records; almost 30% are affiliated with an institution in Europe, ~21% with one in Asia, ~19 and ~2% with North and South America, respectively, while Africa and Oceania account for around ~1% each.

Bibliographic data do not contain information on authors' *gender*, with authors' names being the only piece of information capable of providing a respective indication. We combine responses from different gender assignment services, maximizing the recall (i.e., the number of names that can be assigned a gender), while keeping the error rate under a certain threshold. Our heuristic which is described in Mihaljević and Santamaría (2020) in more detail, is based on a comparison and benchmark (Santamaría and Mihaljevi, 2018) of five dedicated sources for name-based gender inference. In particular, our procedure makes sure that the bias in gender prediction understood as the imbalance of females misclassified as males compared to the error in the opposite direction is kept close to zero. It should be noted that name-based gender inference yields numerous challenges. In addition to accuracy-related issues arising through abbreviations, transliteration etc., it faces, as all approaches for automated gender inference, conceptual and ethical problems such as the usage of a binary scheme (cf. Mihaljević et al., 2019). We would thus like to emphasize that we do not understand “women” and “men” as monolithic categories; our classification is due to pragmatic reasons and, in particular, the lack of data stemming from self-identification.

Finally, we include the year in which a manuscript was published since it is an important predictor of the network size and the overall number of publications, as shown in Figures 1, **5**.

Table 2 summarizes all variables used in the resulting data model.

**Table 2:** Description of all variables in the final dataset, with target variables highlighted in bold.

**Variable**	**Description**	**Computation**	**Example**	**Missing values**
Author ID	Unique zbMATH author identifier		bourbaki.nicolas	No
Year	Publication year		2013	No
Publications	Number of publications, up to and including the respective year	Sum	5	No
**Single-authored publications**	Number of solely written publications, up to and including the respective year	Sum	1	No
Seniority	Number of years since the author's first publication, starting with 0	Count	2	No
**Network size**	Number of distinct collaborators, up to and including the respective year	Count	2	No
Gender	Gender predicted based on name string; binary + “unknown” (cf. Mihaljević and Santamaría, 2020)		Female	~28%
Continent	Continent extracted from the author's earliest available affiliation	First	Asia	~25%
Subfield cluster	Clusters of level-1-codes of the MSC2010, produced by ego-splitting with resolution 1 (cf. Epasto et al., 2017)	Most frequent	Graphs/Linear Algebra	No
Journal ranking	zbMATH's internal processing scheme, reflecting their evaluation of journal's relevance and quality (cf. Mihaljević and Santamaría, 2020)	Most frequent	1	~5%

### 3.2. Data overview

The final dataset comprises 2, 806, 493 records corresponding to 260, 968 unique authors. Among those, 127, 983 are predicted to be male and 34, 793 female.

**Figure 4:**
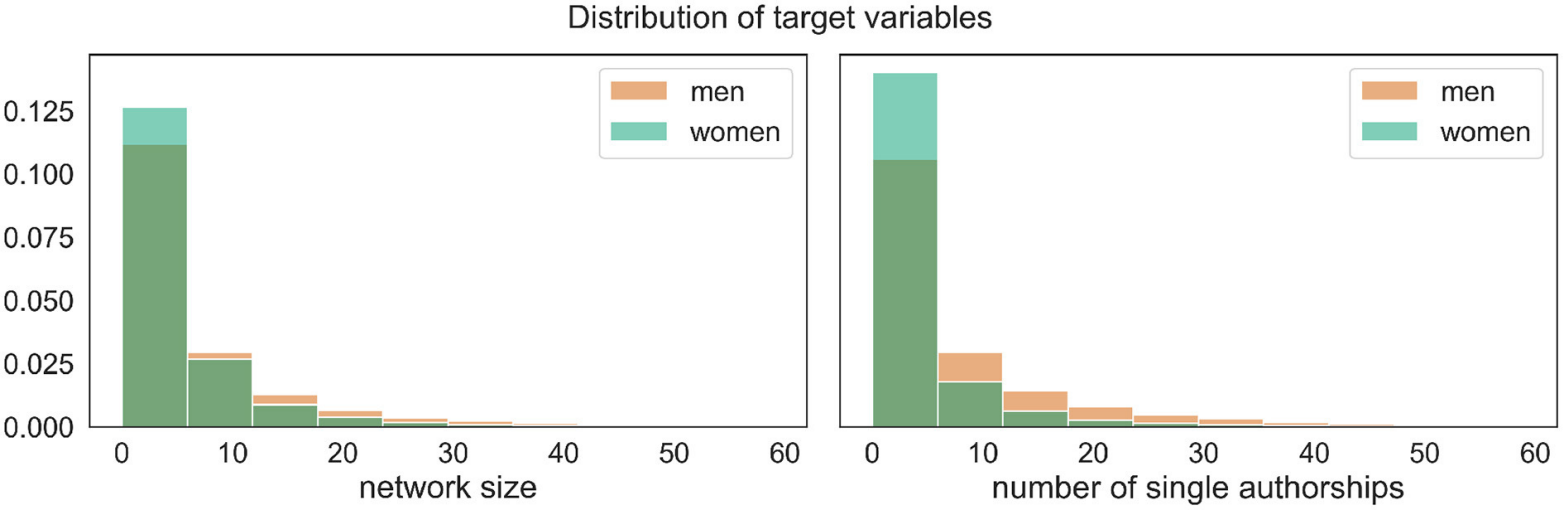
Empirical distributions of the target variables broken down by gender, showing that, without taking any other variables into account, the proportion of women among authors with smaller networks and those writing less papers alone is higher compared to men.

A naıve look at the target variables in relation to gender reveals that men have on average ~6.9 different coauthors (std = 11.5), in contrast to ~4.8 for women (std = 7.7). Similarly, men write almost eight publications alone (std = 13.9), while women have ~3.4 solo-authored articles (std = 13.9). In terms of percentages, this translates into an average of around 41% of publications written alone among men vs. 30% among women. Figure 4 displays the empirical distributions of both target variables.

**Figure 5:**
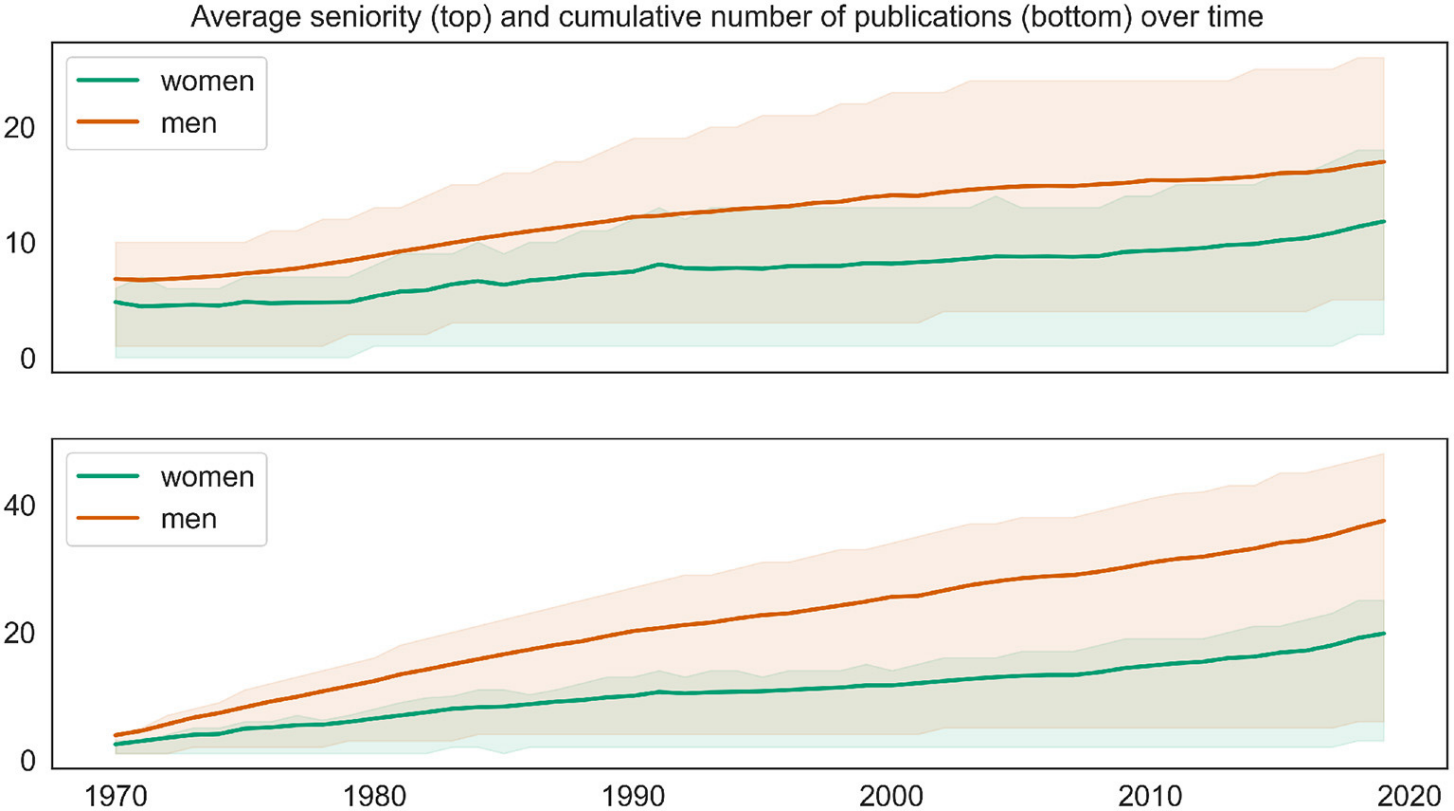
Average author seniority **(top)** and number of publications **(bottom)** accumulated until a given year, broken down by authors' gender. Shaded areas mark the [25, 75] confidence intervals; in the more saturated shaded area confidence intervals for women and men overlap.

However, these numbers alone are misleading because the two groups differ significantly with respect to the most important factors regarding the target variables, namely the number of publications and seniority (cf. Figure 1). Male mathematicians publish more, and the proportion of senior men is significantly higher, as Figure 5 demonstrates.

### 3.3. Methods

**Figure 6:**
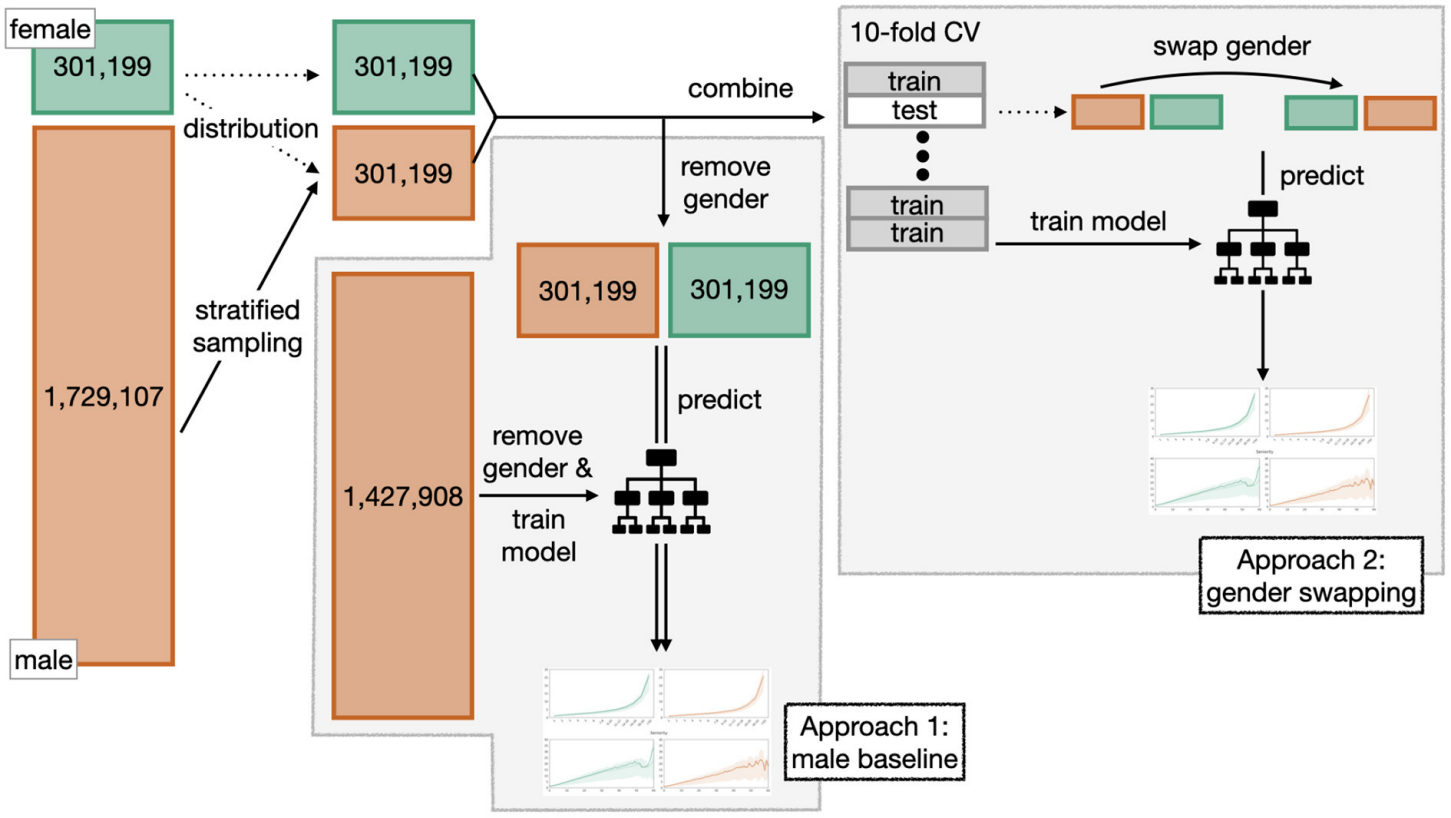
Schematic illustration of the two approaches “male baseline” and “gender swapping” used to isolate and quantify the effect of gender.

To isolate and quantify the effect of gender on each of the target variables, we adapt two known techniques and apply them in different variations to ensure robustness. A schematic illustration of both methods can be seen in Figure 6.

#### 3.3.1. Stratified random sampling

Since the dataset contains a lot more records for men than for women that show highly differing distributions w.r.t. multiple features, we need a way to generate two comparable datasets for women and men to produce meaningful results. We generate two equally sized datasets using stratified sampling, so that per stratum, the number of records from men and women are equal.

**Figure 7:**
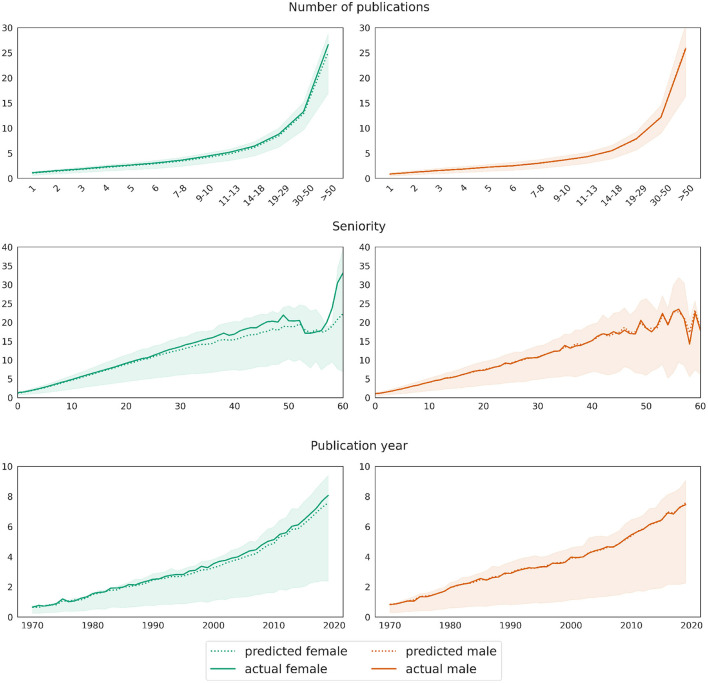
Male baseline approach: mean differences between actual (solid curve) and predicted (dotted curves) values for the target variable *network size*. Shaded areas mark the [25, 75] confidence interval of the ground truth data.

In our case, it makes sense to choose either the number of publications or the seniority as stratification variable, because these two have the highest correlation with our target variables (see Figure 1). However, since the number of publications reveals a long tail distribution, we first group the values into 13 segments between 1 and >50, where in particular higher numbers of publications are merged together (see, e.g., the x-scale in Figure 7: Number of publications). Due to the size imbalance we sample from the subset of records representing men based on the number of records of women per stratum.

#### 3.3.2. Approach 1: Male baseline

We follow the approach of Caplar et al. (2017) who model the effect of gender on the number of citations and authorships in prestigious astronomy and astrophysics journals. For each of the two target variables we train a prediction model that does not take the gender variable as a feature, and is trained using only records from male mathematicians. We then apply the trained model to test data sets from both women and men separately and evaluate the differences between true and predicted values in each case. This lets us explore how women would be seen by an oracle that only knows men, and compare it to the real world. For this approach, the female test set consist of all 301,199 female records. The equally sized male test set is created using the stratified sampling method described above. The remaining 1,427,908 male records comprise the training set.

#### 3.3.3. Approach 2: Gender swapping

Our second approach is inspired by the idea from the experiment of Bertrand and Mullainathan (2004) who sent resumes for job ads in order to measure the effect of attributes such as gender and race on the likelihood to get invited for an interview. For each target variable, we train a prediction model using data from both women and men, including gender as one of the features. We make sure that the two datasets are balanced in terms of gender by using the stratification described above to sample an equal amount of male records. This yields a dataset of ~600,000 records. Within a 10-fold cross-validation we train models and compute scores on test sets. In each round, the training data comprises 90% (542,158 records) and the test set 10% (60,240 records) of the dataset, both balanced in terms of gender.

In addition, we swap the gender attribute in the test data for their complement and recompute the scores. The swapping helps to infer how the target variable would be predicted if only the gender was different but everything else stayed the same.

#### 3.3.4. Variations

We aim for tree-based prediction models since they are well capable of capturing nonlinear relationships. The optimal models are found using randomized hyperparameter search. To make sure that the modeling and the resulting evaluation are as robust as possible, we implement different variants of the two approaches using combinations of the following parameters:

- Stratify by number of publications and seniority and run each on multiple randomly chosen data samples.
- Use Gradient Boosting Regression (Friedman, 2001) and Random Forest Regression (Breiman, 2001) as algorithms.
- Evaluate models with different hyperparameters: in addition to the model with optimal hyperparameters, evaluate another model that causes less overfitting but still shows superior performance on the validation set.
- In the first approach, use the entire training data consisting of 1,427,908 records, or apply the same stratification used for creating the test set to sample 791,937 records with a similar distribution.

## 4. Results

All evaluations are implemented in Python 3.6 using the scikit-learn library (version 0.24.2; Pedregosa et al., 2011). The different variants yield very similar results, showing that our procedure is reliable and robust. Thus, here we only report the results from training a GradientBoostingRegressor^3^ with optimal hyperparameters and the number of publications as the stratification variable (For the first approach male training data is not additionally sampled). Since the setting between both approaches is very similar, we apply the hyperparameter search only within the first approach and reuse the results for the second. The hyperparameter search yields 140 estimators, a minimum of 5 samples per leaf and a maximum depth of 15 as optimal parameters for both target variables. Code and evaluations covering all implemented scenarios can be found in a public repository (https://github.com/math-collab/gender).

### 4.1. Network size

For the first approach, our best performing regression achieves a mean training score of 0.85 and a mean test score of 0.74. As expected from the explorations in Section 3.1, the most important feature is the number of publications (relevance score: 0.75), followed by author seniority (0.07) and the publication year (0.06). The model yields *R*^2^ scores for test data of 0.63 (female) and 0.68 (male).

As explained previously in Section 3.3, we evaluate the model on two test sets for men and women, respectively, each consisting of 301,199 records. The model underestimates the number of coauthors for women: while the mean difference between predictions ŷ and true values *y* for the male test set is 0.001, it equals −0.227 on the female test set. The plots in Figure 7 illustrate the deviation between the model's prediction and the ground truth for both test sets, dis-aggregated by the number of publications, author seniority and the publication year. The curve representing the real values overlaps almost perfectly with the curve representing predicted values on the male test set. A *t*-test for two related samples shows no significance between real and predicted data for men in any of the groups reflecting the number of publications. This confirms the prediction quality of the model on the male test data. On the female test set, the curve representing predicted values lies below the one representing true values, and the differences between real and predicted values per any of the groups are significant (*p*-value 0.01). The divergence increases with growing number of publications, higher seniority and latter publication years. However, it should be noted that the number of records in the respective segments is a lot smaller, as, e.g., there are rather few women with a seniority of 50 years or above. Thus, the deviation between real and predicted values for women can be seen as rather low, meaning that women exhibit in fact slightly larger coauthor networks if we control for variables such as the number of publications, seniority, subfield cluster etc.

**Figure 8:**
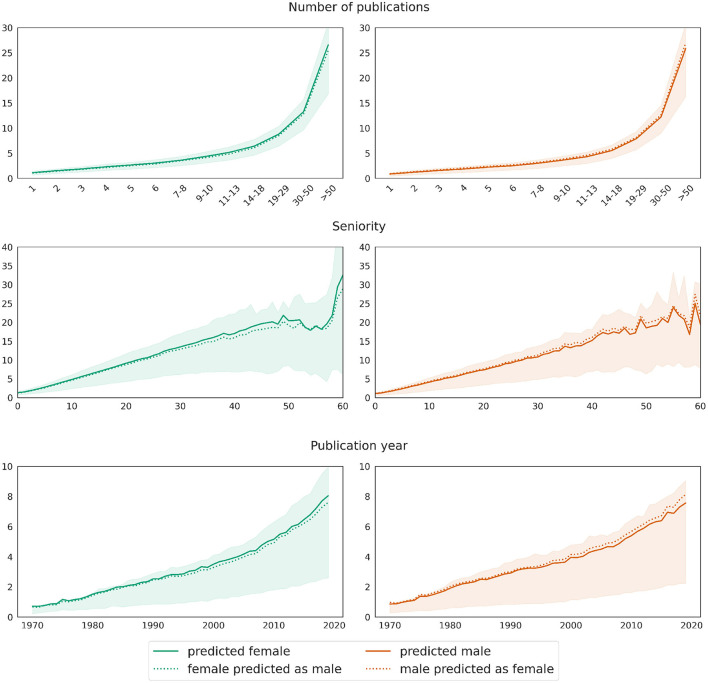
Gender swapping approach: deviations in the value distributions of predictions applied to real data (solid curves) and data with swapped gender values (dotted curves) for the target variable *network size*. The curves show mean values, while shaded areas mark the [25, 75] confidence interval of the predictions on real test data.

Our second approach, yielding comparable train and test scores, confirms these observations. Figure 8 shows in the left column the predictions for the female test set as a solid curve and those for the same data but with swapped gender value from female to male as a dotted curve. Similarly, the results for the male test set are displayed in the right hand column. Again, the visualizations reflect the decomposition of the predictions by the number of publications, the author seniority and the publication year. The plots show that the predicted number of coauthors slightly decreases for female mathematicians when changing their gender from female to male in the test set. This tendency is exactly the opposite for male mathematicians in the test set who show a slight increase when swapping their gender to female. Again, the deviations between the real and the swapped data increase with growing number of publications, seniority, and for later publication years.

### 4.2. Number of single authorships

Using the first approach, we obtain very similar performance results on training and test data (mean train score of 0.87 and mean test score of 0.78) as for network size as target variable. Again, the number of publications has the largest influence on the model, but slightly less than before (relevance score: 0.66), followed at a large distance by author seniority (0.08), and publication year (0.07). The model, trained on data representing men's publications, is significantly less able to explain the variance in the female than in the male test set, with *R*^2^ scores of 0.53 and 0.79, respectively. This already indicates that there is a significant difference between the statistical properties of the two datasets.

The evaluation of the predictions reveals this difference in more detail: The male-baseline model overestimates the number of papers written alone by women, and this difference is statistically significant for each of the groups (*t*-test for paired samples with *p*-value 0.01). The model predicts women to have around half a publication more written alone (mean difference between ŷ and *y* for the female test set is 0.55), while being almost zero (−0.004) for the male test set; the difference between real and predicted values is not statistically significant for any of the groups of men's publications.

**Figure 9:**
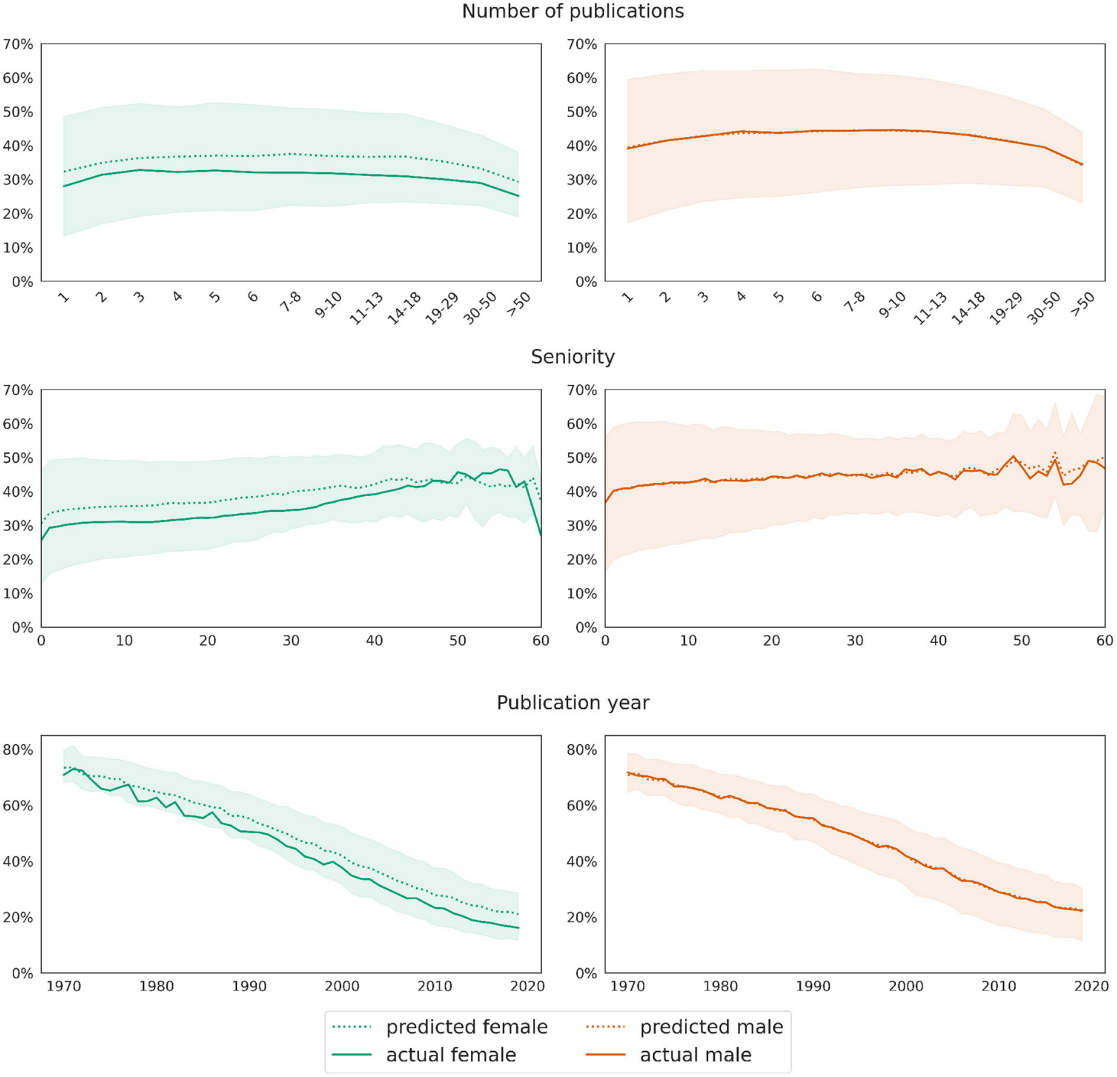
Male baseline approach: Mean differences between actual (solid curve) and predicted (dotted curves) values for the target variable *number of single authorships*. Shaded areas mark the [25, 75] confidence interval of the ground truth data.

As for the previous model, we visualize the difference between predictions and the ground truth for both test sets in Figure 9. In order to highlight the differences between the distributions, the diagrams show the percentages of single authorships among the total authorships instead of counts. As before, the data is broken down by the number of publications, seniority, and year, respectively. In contrast to the prediction of network size, the difference between women and men in terms of the number of single authorships is not only significantly larger, but also stable across the respective numbers of publications, seniorities, and publication years. As Figure 9 shows, the mean percentage of single authorships of women is around 4–5% (on average 4.6%) less than predicted by the baseline male model, which is significantly less than the difference of ~11% observed in the raw data without taking into account any other variables (see Section 3.2).

**Figure 10:**
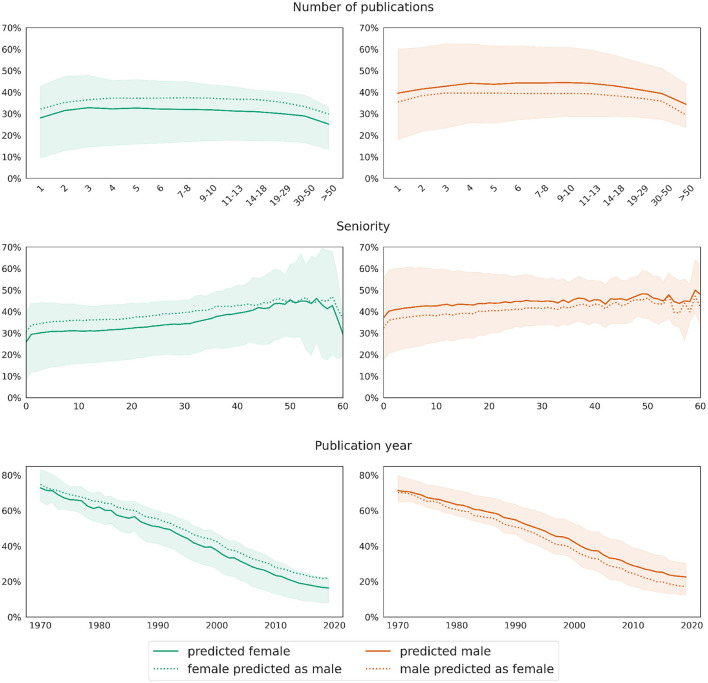
Gender swapping approach: deviations in the value distributions of predictions applied to real data (solid curves) and data with swapped gender values (dotted curves) for the target variable *number of single authorships*. The curves show mean values, while shaded areas mark the [25, 75] confidence interval of the predictions on real test data.

Figure 10 displays the average predictions obtained from models trained within a 10-fold cross-validation on data balanced in terms of gender and number of publications and applied to test data with correct and swapped gender values. As before, this approach yields results analogous to the previous one, confirming the robustness of our overall approach. More precisely, the upper row of Figure 10 illustrates well that the model predicts women to have around 11% less authorships written alone than men, and this difference remains stable across all bins. However, for the most part, the difference is due to other variables; as shown by a comparison between the solid and dashed curves in the respective subplots, the prediction deviates by around 4.5% upward (swapping from female to male) or downward (swapping from male to female) when the value for the gender variable in the respective test data set is swapped.

The first rows in both Figures 9, 10, respectively, additionally show that the proportion (as opposed to the total number) of single authorships depends little on the total number of publications. The curves for both genders are relatively horizontal across most segments, with a slight dip at the beginning and end. At the same time, the bottom row of both plots reveals the evolution of the discipline away from single authorships toward more collaboration. This trend is also reflected in the middle row of the graphs, where the curves rise slightly with increasing seniority of authors.

#### 4.2.1. Local interpretations using SHAP values

To illustrate how a machine learning model utilizes the main features, especially the author's gender, to arrive at a prediction of the number of single authorships, we train a Gradient Boosting classifier and apply SHAP (Lundberg and Lee, 2017), a *post-hoc* explanation technique for machine learning models. We train the model based on the 602, 398 records as displayed in Figure 6 (the data is balanced in terms of author's gender) but this time including the gender variable. We make use of the Python SHAP package^4^ that assigns to every input feature a relevance score for each prediction. The scores, which are computed using Shapley values from cooperative game theory, can be used as local interpretations for individual predictions, showcasing a model's reasoning for a particular data record.

For the local comparisons, we create personas with seniority levels of 5, 10, and 15 years, respectively, that represent different career stages: while a seniority of 15 years can be associated with a secure permanent academic position, 5 years can rather be seen as a realistic proxy for an early postdoctoral stage in mathematics. We also fix the number of publications per seniority level as 3, 6, and 10, respectively. To showcase the effect of different mathematical subfields, we additionally differentiate between the cluster “PDE/Numerical/Physics,” an interdisciplinary and applied cluster, and “Number theory/Algebraic geometry,” a combination of two rather traditional areas of pure mathematics. In addition, we create a female and male version of each combination, yielding twelve personas altogether. We fix the remaining features by setting 2010 as the publication year, North America as the continent and journal rank to be 1.

**Figure 11:**
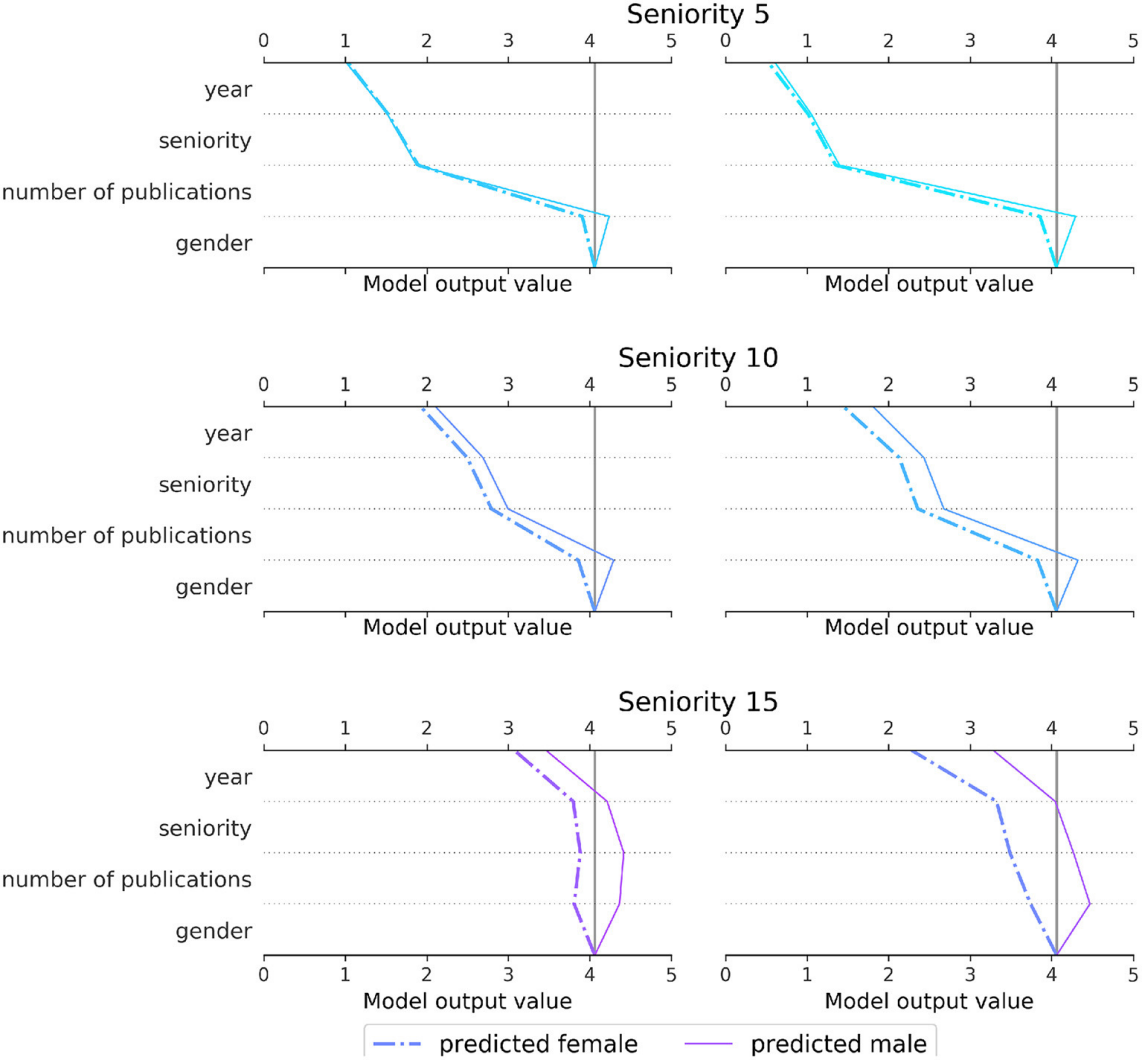
SHAP decision plot of a Gradient Boosting classifier predicting the number of single authorships for twelve different personas. We display the local relevance of the four main features “year,” “seniority,” “number of publications,” and “gender.” The curves representing the SHAP values are relative to the model's expected value which is approximately four in this case. The SHAP values for each feature are successively added to the expected value of the model (viewed from the bottom up), showing how each feature contributes to the overall prediction. The rows represent different seniority levels (5, 10, and 15), the columns correspond to the subfield clusters “PDE/Numerical/Physics” **(left)** and “Number theory/Algebraic geometry” **(right)**, while the line types differentiate between the persona's gender. We assumed the continent to be North America, the publication year to be 2010 and the dominating journal rank to be 1.

Figure 11 displays the relevance scores of the main features of the model for each of the twelve personas, with columns representing the subfield clusters, rows the seniority levels and line types the author's gender. In all six plots it can be seen that the gender variable causes the two curves to diverge, with the dashed curve representing the female persona moving further to the left. This shows that the model associates women (for the personas shown here) with a lower number of single authorships. This effect is partially mitigated by other variables: especially for the personas with seniority 5 (first row), the difference is nearly neutralized by the variable representing the total number of publications. A likely reason for this is our assumption of the same number of publications for both genders at the same seniority. Since male mathematicians tend to have a larger output of publications (Mihaljević and Santamaría, 2020), the parameters for the two genders are associated with different frequencies, so the model assigns a stronger positive weight to the total number of publications for the female persona. Furthermore, the deviation between the two curves per plot increases with seniority for both subfield clusters, though more or less in proportion to the absolute number of publications, confirming the overall tendency captured in Figure 10.

## 5. Summary and discussion

Successful research careers typically involve both individual and collaborative efforts. The persistence of gender differences in mathematics as a research discipline, not only at the level of simple counts but in the successful shaping of careers, necessitates an examination of corresponding collaborative practices.

To assess the role of gender on the network size and the amount of single authorships, we have applied two approaches that allow to separate the effect of gender from that of other gender-correlated variables. In the “male baseline” approach, a predictive model is trained on male data and the evaluations on female and male test data were compared. In the “gender swapping” approach, we have trained predictive models on data from men and women and applied them to test data with real and swapped values for the gender variable. We have shown that women have even slightly larger networks when controlling for total number of publications, seniority, publication year, subfield, continent of work, or perceived journal quality. Under the same modeling conditions, women have fewer single authorships, though the difference is smaller than previous work has suggested, at 4.5% compared to men. It follows that with respect to the two dimensions of network size and number of single authorships, there are rather small gender-specific differences, and these alone can presumably contribute little to the explanation of the gender gap in mathematics.

In addition, we have trained a model to predict the number of solo authorships, taking into account the gender variable as well, and illustrated its reasoning by applying SHAP decision plots to predictions for twelve personas. We provide the trained model together with code for the computations of SHAP decision plots in the project's Github repository to enable other researchers to explore the predictions performed by the model in more detail.

In addition to pure network size, there are a number of other collaboration-related parameters, such as gender or seniority of coauthors, ranking of affiliated university, frequency of co-authorship or connectivity within the ego-network, which we do not investigate in more detail. For other disciplines, not only correlations of such aspects with performance but also gender-related differences are known (Bozeman and Gaughan, 2011; Lindenlaub and Prummer, 2016; Jadidi et al., 2018; Ductor et al., 2021; Kwiek and Roszka, 2022). Future work could investigate corresponding questions for mathematics. However, some aspects such as university-level considerations require the extraction of respective named entities from affiliation strings and a significantly higher availability of affiliation data.

We focus on coauthorship as the main form of documenting collaboration in mathematics (and most other sciences). Although other forms of collaboration, such as sharing ideas or giving feedback at seminars or conferences, play an important role in scientific work in general, coauthorship is, through databases such as zbMATH Open, a comprehensively available, measurable, and creditable form of contribution and therefore not only important for scientific careers, but also particularly suitable from a methodological point of view. Nevertheless, other, less formal types of scientific collaboration deserve a closer look but typically lack data that can be utilized for respective analyses. However, recently, acknowledgments in scholarly articles that serve as a form of credit attribution were analyzed in more detail, suggesting that corresponding practices are associated with academic status and gender (Paul-Hus et al., 2020). More in-depth analyses would also be of interest for the field of mathematics.

## Data availability statement

The datasets presented in this study can be found in online repositories. The names of the repository/repositories and accession number(s) can be found at: https://github.com/math-collab/gender.

## Author contributions

All authors listed have made a substantial, direct, and intellectual contribution to the work and approved it for publication.

## Footnotes

^1^An authorship is assigned to an author ID *via* a combination of algorithmic procedures and manual checking by zbMATH staff. Like any disambiguation procedure, it is not perfect and can both split and incorrectly augment author profiles. However, due to the enormous effort invested into the development of their author name disambiguation procedures, and especially the involvement of the mathematical community in the correction of author profiles via a dedicated web interface, a solid state has been reached in the meantime. For more details on zbMATH's creation of author profiles, see Mihaljević-Brandt et al. (2014) and Müller et al. (2017).

^2^ https://www.msc2010.org/mediawiki/index.php?title=MSC2010

^3^ https://scikit-learn.org/0.24/modules/generated/sklearn.ensemble.GradientBoostingRegressor.html

^4^ https://github.com/slundberg/shap
